# Driving pressure and mortality in trauma without acute respiratory distress syndrome: a prospective observational study

**DOI:** 10.5935/0103-507X.20210033

**Published:** 2021

**Authors:** Jairo Corrêa da Silveira Júnior, Eder Kröeff Cardoso, Marcelo de Mello Rieder

**Affiliations:** 1 Centro Universitário Metodista IPA - Porto Alegre (RS), Brazil.; 2 Hospital de Pronto Socorro de Porto Alegre - Porto Alegre (RS), Brazil.

**Keywords:** Respiratory distress syndrome, adult, Trauma, Mortality, Respiration, artificial, Respiratory mechanics, Critical care, Intensive care units, Síndrome do desconforto respiratório do adulto, Trauma, Mortalidade, Respiração artificial, Mecânica respiratória, Cuidados intensivos, Unidades de terapia intensiva

## Abstract

**Objective:**

To identify the possible association between driving pressure and mechanical power values and oxygenation index on the first day of mechanical ventilation with the mortality of trauma patients without a diagnosis of acute respiratory distress syndrome.

**Methods:**

Patients under pressure-controlled or volume-controlled ventilation were included, with data collection 24 hours after orotracheal intubation. Patient follow-up was performed for 30 days to obtain the clinical outcome. The patients were admitted to two intensive care units of the *Hospital de Pronto Socorro de Porto Alegre* from June to September 2019.

**Results:**

A total of 24 patients were evaluated. Driving pressure, mechanical power and oxygenation index were similar among patients who survived and those who died, with no statistically significant difference between groups.

**Conclusion:**

Driving pressure, mechanical power and oxygenation index values obtained on the first day of mechanical ventilation were not associated with mortality of trauma patients without acute respiratory distress syndrome.

## Abstract (pt)

**Objetivo:**

Identificar a existência de associação entre os valores de *driving pressure e mechanical power* e do índice de oxigenação no primeiro dia de ventilação mecânica com a mortalidade de pacientes vítimas de trauma sem diagnóstico de síndrome do desconforto respiratório agudo.

**Métodos:**

Foram incluídos pacientes ventilados em modo de pressão ou volume controlado, com coleta de dados 24 horas após sua intubação orotraqueal. O acompanhamento do paciente foi realizado por 30 dias para obter o desfecho clínico. Os pacientes estiveram internados em duas unidades de terapia intensiva do Hospital de Pronto Socorro de Porto Alegre, no período de junho a setembro de 2019.

**Resultados:**

Foram avaliados 24 pacientes. Os valores de *driving pressure, mechanical power* e do índice de oxigenação foram similares entre os pacientes que sobreviveram e os que tiveram desfecho de óbito, sem diferença estatisticamente significativa entre os grupos.

**Conclusão:**

Os valores de *driving pressure, mechanical power* e índice de oxigenação obtidos no primeiro dia de ventilação mecânica não demonstraram ter associação com a mortalidade de pacientes vítimas de trauma sem síndrome do desconforto respiratório agudo.

## INTRODUCTION

Trauma has increased exponentially as one of the main causes of death. This fact is associated with the increase in urban violence, which occurs due to various socioeconomic factors, and with technical developments in the automotive industry, which is increasingly able to produce vehicles with greater motor power, which are more likely to cause serious accidents. Such an association is demonstrated in epidemiological data, which indicate that traffic accidents are the fifth leading cause of death worldwide.^(1,2)^

Trauma leads to a rapid and complex response of the affected body, whose immune homeostasis is disturbed, leaving the individual vulnerable to infections and inflammatory complications. This response is influenced by the type and severity of the trauma, as well as aspects of the individual, such as sex and age, among other factors.^(3)^

Trauma cases are more frequent in low and middle incomes countries, which account for the majority of the world’s population. In these developing countries, armed conflicts occur more frequently, and there is a high prevalence of accidents involving motor vehicles. In addition, trauma occurring in these countries tends to be more severe, resulting in clinically severe patients.^(4)^

In critically severe patients, there is a need to protect the airway and maintain gas exchange, which is achieved through mechanical ventilation, which is present in all intensive care units (ICUs) and provided as total or partial assistance, to ensure gas exchange and maintain adequate levels of oxygen and carbon dioxide in the blood. However, complications arise from the use of mechanical ventilation, for example, ventilation-associated pneumonia and ventilation-induced diaphragmatic dysfunction.^(5,6)^

Several variables influence clinical outcomes and have been gradually implemented to monitor the ventilatory management of patients on mechanical ventilation; these variables include mechanical power, the oxygenation index and driving pressure (DP). Among these, DP has received greater attention with respect to protective ventilation. Its importance has increased based on the identification of its correlation with the survival of patients with acute respiratory distress syndrome (ARDS) under controlled mechanical ventilation (MV).^(7)^

Thus, the objective of this study was to identify possible associations between DP, mechanical power and oxygenation index values on the first day of MV with the mortality of trauma patients without an ARDS diagnosis.

## METHODS

This was a prospective observational study conducted in the ICUs of the *Hospital de Pronto Socorro de Porto Alegre* from June to September 2019. The sample size was calculated based on the study by Schmidt et al., which included 622 individuals, with a 95% confidence level and 20% margin of error. The sample size was calculated in accordance with Levine et al.^(8,9)^

The study included male and female patients aged ≥ 18 years who victims of external trauma and mechanically ventilated by an orotracheal tube in pressure-controlled ventilation (PCV) or volume-controlled ventilation (VCV) mode. Patients who remained under controlled ventilation for less than 24 hours were excluded from the study. Thus, 26 patients were initially included, with two subsequently excluded. This study complies with resolution 466/12 and was approved by the Research Ethics Committee of the IPA Methodist University Center, under number 3.498.318.

The participants’ guardians were invited to sign an informed consent form, authorizing the participation of the individual. Descriptive data were collected, always by the same evaluator, at the time of selection in a specific evaluation form containing identification data.

Bedside data collection began 24 hours after the time of intubation reported in the electronic medical record. The variables fraction of inspired oxygen (FiO_2_), positive end-expiratory pressure (PEEP), respiratory rate (*f*), expired tidal volume (VT), peak pressure (Ppeak) and plateau pressure (Pplat) were collected on a Servo-S mechanical ventilator (MAQUET Critical Care AB, Solna, Sweden). To obtain Pplat, an inspiratory pause of at least 2 seconds was performed, in accordance with the Brazilian Mechanical Ventilation Recommendations.^(10)^

After recording these data, the oxygen partial pressure (PaO_2_) value from the arterial blood gas analysis performed on the same day was collected. Using the PEEP and Pplat values, the DP in cmH_2_O (Pplat-PEEP) was obtained; for the PaO_2_ and FiO_2_ values, the oxygenation index was obtained; and for the *f*, VT, Ppeak and DP values, the pulmonary mechanical power (MP) was obtained in J/minute (0.098 x *f* x VT x (Ppeak - DP/2).^(11,12)^

Participants were monitored through their electronic medical records, from which the total length of stay on MV and clinical outcome in the hospital were obtained.

The Statistical Package for Social Sciences (SPSS), version 20.0, was used for statistical analysis. Descriptive statistics were used for quantitative data (mean ± standard deviation) and categorical data (absolute frequency). Quantitative data were compared by Student’s t-test for independent data. Subsequently, logistic regression analysis was performed with the outcome as a dependent variable. A significance level of p < 0.05 was adopted in all tests.

## RESULTS

A total of 24 individuals were included in the study between June and September 2019 from two ICUs of the *Hospital de Pronto Socorro de Porto Alegre*. Most patients were male (87.5%). The predominant ventilation mode was PCV (83.3%). The other characteristics of the sample are provided in table 1.

**Table 1:** Clinical characterization of the sample

Variables	
Age (years)	42 ± 18
Sex (f:m)	3:21
Duration of mechanical ventilation (days)	9 ± 6
Mechanical ventilation mode	
PCV	20
VCV	4
PaO2 (mmHg)	119 ± 38
Oxygenation index	305 ± 107
PEEP (cmH_2_O)	6 ± 1
Ppeak (cmH_2_O)	21 ± 4
Driving pressure (cmH_2_O)	12 ± 3
Mechanical power (J/minute)	15 ± 6
Type of trauma	
TCE	9
Chest trauma	2
Face trauma	4
Polytrauma	8
SCT	1
Death	6

f - female; m - male; PCV - pressure-controlled ventilation; VCV - volume-controlled ventilation; PaO_2_ - partial pressure of oxygen; PEEP - positive end-expiratory pressure; Ppeak - peak pressure; TBI - traumatic brain injury; SCT - spinal cord trauma. The results are expressed as the mean ± standard deviation or absolute frequency.

**Figure 1:**
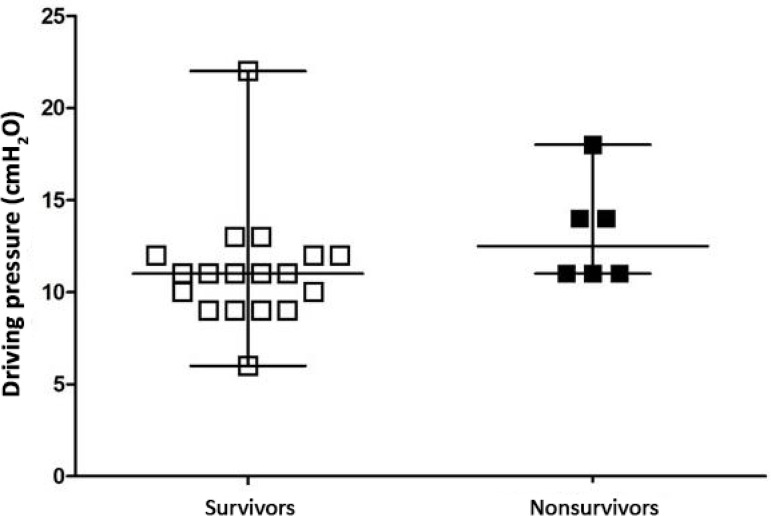
Evaluation of driving pressure in survivors and nonsurvivors. Student’s t-test for independent data (p = 0.80).

**Figure 2:**
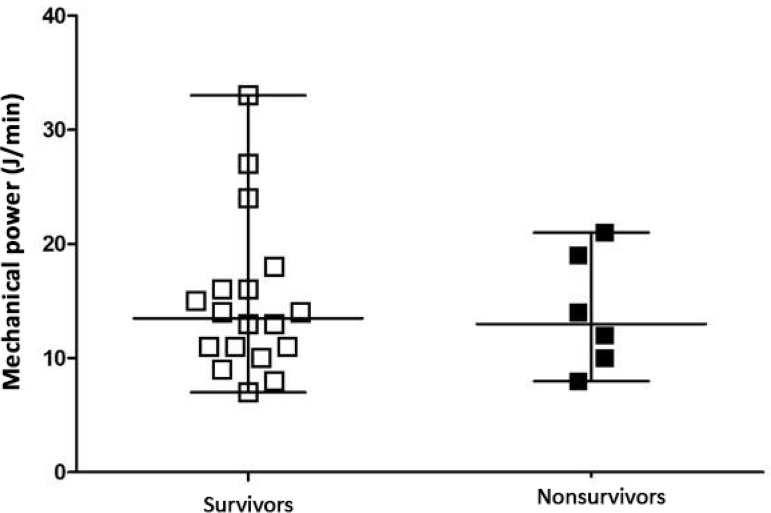
Evaluation of mechanical power in survivors and nonsurvivors. Student’s t-test for independent data (p = 0.66).

**Figure 3:**
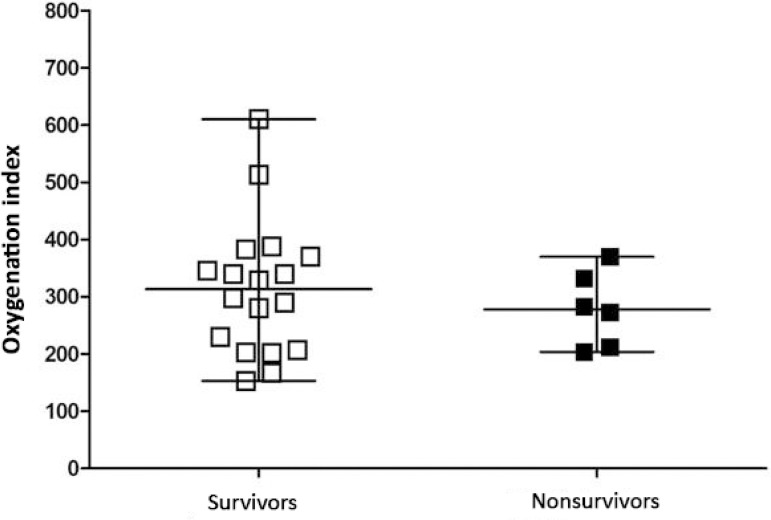
Evaluation of the oxygenation index in survivors and nonsurvivors. Student’s t-test for independent data (p = 0.23).

Figures 1 to 3 show the distribution of DP, MP, and oxygenation index values between the surviving and nonsurviving groups, respectively. There was no statistically significant difference between groups for DP (p = 0.8), MP (p = 0.66) or oxygenation index (p = 0.23) values.

Logistic regression analysis was performed with the dependent variable being “death”. The variables DP (odds ratio - OR = 1.67; 95% confidence interval - 95%CI = 0.87 - 3.22; p = 0.12), MP (OR = 0.80; 95%CI = 0.58 - 1.12; p = 0.2) and oxygenation index (OR = 0.998; 95%CI = 0.98 - 1.01; p = 0.7) were not predictors of death in the sample.

## DISCUSSION

Most patients admitted to the ICU were male, with a MV duration > 7 days, and traumatic brain injury was the most common type of trauma (37.5%), a finding that is consistent with the literature.^(13)^

In this study, the DP, MP and oxygenation index values on the first day of MV were not associated with the clinical outcome of trauma victims without ARDS. These findings suggest that in the studied population, initial ventilatory management may not be an aggravating factor in clinical prognosis.

Other studies have sought to identify the association of DP with hospital mortality in other populations without ARDS. Simonis et al. evaluated 935 patients, identifying that the survivors had lower DP, PEEP, FiO_2_ and Ppeak values. However, DP was not independently associated with hospital mortality.^(14)^ This result was similar to that reported by Schmidt et al., who evaluated 622 patients on the first day of MV to identify an association between DP and mortality in patients without ARDS, with no association with hospital mortality.^(8)^

Regarding the association between PDPD and mortality, De Ferrari et al. analyzed data from 1,212 patients on the first day of MV. In that sample, DP was associated with the mortality of patients with and without a diagnosis of ARDS within 90 days after hospital discharge.^(15)^ In turn, Sahetya et al. found that in a sample of 1,132 patients on MV with and without ARDS, higher DP values were associated with higher hospital mortality in patients without ARDS.^(16)^

Fuller et al. evaluated DP in 1,705 patients on the first day of ventilation to assess association with mortality and the incidence of ARDS. Among those patients, 152 developed ARDS and had significantly higher DP values. Patients who died also had significantly higher DP values, which was independently associated with hospital mortality.^(17)^

However, an ARDS diagnosis is delayed or missed in two-thirds of patients.^(18)^ The scientific findings obtained thus far are controversial regarding the real association between the ventilatory parameters studied and the mortality of the population without ARDS. This high diagnostic miss rate may influence the results when studying this population.

Other studies evaluated the association between the oxygenation index and mortality in the first 48 hours of MV. Whiting et al. evaluated 281 individuals, identifying an association between oxygenation index values < 100, compared with oxygenation index values > 500, and increased mortality, demonstrating a trend toward higher mortality in patients with severe gas exchange impairment. In our sample, the values between survivors and nonsurvivors were similar, with no significant difference between them.^(19)^

Analyzing MP and its association with mortality, Fuentes Goméz et al. included 67 patients in their study, comparing values on the first and third days of MV. The findings for MP on the first day of MV were similar to those found in our study, with no difference between the values for survivors and nonsurvivors. However, MP values on the third day of MV were associated with higher mortality.^(20)^ Serpa Neto et al. analyzed data from 8,207 mechanically ventilated patients for at least 48 hours, identifying that higher MP values were independently associated with higher hospital mortality, showing a consistent increase in the risk of death with MP greater than 17 J/minute. This may indicate that the concept of ergotrauma presented by Tonetti et al. has a cumulative influence on ventilatory management.^(21.22)^

The existing evidence on the evaluated parameters does not yet fully clarify their real importance in the study population or a need for constant monitoring in clinical practice. Among the limitations of this study, the small sample size may be a factor that influenced the obtained results. Future studies should restrict the study population to obtain a homogeneous cohort for analysis.

## CONCLUSION

Driving pressure, mechanical power, and oxygenation index values on the first day of mechanical ventilation were not associated with the mortality of trauma patients without acute respiratory distress syndrome.
